# Perforated duodenal tuberculosis with pancreaticobiliary disruption requiring salvage pancreaticoduodenectomy: a case report

**DOI:** 10.1093/jscr/rjag801

**Published:** 2026-09-03

**Authors:** Mirko Lombardi, Tulia Hadi, Bahati Kamwezi, Lugano Wilson, Lusekelo Mwalukambwe, Edrick Elias, Tresphory Boniphace Kamushaga, Shama Mkini, Agnes Mlawa

**Affiliations:** Department of Surgery, Consolata Hospital Ikonda, Makete District, Njombe Region, PO Box 700, Njombe, Tanzania; Department of Surgery, Consolata Hospital Ikonda, Makete District, Njombe Region, PO Box 700, Njombe, Tanzania; Department of Surgery, Consolata Hospital Ikonda, Makete District, Njombe Region, PO Box 700, Njombe, Tanzania; Department of Intensive Care, Consolata Hospital Ikonda, Makete District, Njombe Region, PO Box 700, Njombe, Tanzania; Department of Intensive Care, Consolata Hospital Ikonda, Makete District, Njombe Region, PO Box 700, Njombe, Tanzania; Department of Pathology, Consolata Hospital Ikonda, Makete District, Njombe Region, PO Box 700, Njombe, Tanzania; Department of Surgery, Consolata Hospital Ikonda, Makete District, Njombe Region, PO Box 700, Njombe, Tanzania; Department of Surgery, Consolata Hospital Ikonda, Makete District, Njombe Region, PO Box 700, Njombe, Tanzania; Department of Surgery, Consolata Hospital Ikonda, Makete District, Njombe Region, PO Box 700, Njombe, Tanzania

**Keywords:** duodenal tuberculosis, duodenal perforation, pancreaticoduodenectomy, pancreaticobiliary disruption, emergency surgery, case report

## Abstract

Gastroduodenal tuberculosis is uncommon, and duodenal perforation is exceptional. A 25-year-old man from rural Tanzania presented with generalized peritonitis after prolonged epigastric symptoms, weight loss, night sweats, and household tuberculosis exposure. Emergency laparotomy showed destructive pyloroduodenal perforation with insufficient viable tissue for local repair; distal gastrectomy and Roux-en-Y gastrojejunostomy were performed. Persistent high-output pancreatic-type drainage and progressive jaundice prompted relaparotomy, which demonstrated pancreaticoduodenal destruction and loss of distal biliary continuity. A tailored salvage pancreaticoduodenectomy was required. Histology showed necrotizing granulomatous duodenitis extending into peripancreatic fat and gallbladder serosa while sparing pancreatic acini and gallbladder mucosa. Antituberculous treatment was started, and the patient was well at 3 months. Pancreaticoduodenectomy is not standard treatment for duodenal tuberculosis but may exceptionally be unavoidable when lesser procedures cannot restore source control and anatomical continuity.

## Introduction

Gastroduodenal tuberculosis accounts for <2% of gastrointestinal tuberculosis and usually presents with non-specific epigastric pain, vomiting, weight loss, upper gastrointestinal bleeding, or gastric outlet obstruction [1–3]. Perforation is exceptional, and diagnosis may be delayed until surgery or histopathological examination. Published perforated duodenal tuberculosis cases were managed with local repair, ulcer excision, or other limited procedures [4–6]. By contrast, pancreaticoduodenectomy associated with tuberculosis has generally been performed when pancreatic, periampullary, or gastroduodenal disease mimicked malignancy [7–10]. We report a different sequence: destructive perforating duodenal disease with contiguous extension into adjacent tissues, followed by pancreaticoduodenal disruption and loss of distal biliary continuity. Pancreaticoduodenectomy became necessary for anatomical failure rather than suspected cancer.

## Case report

A 25-year-old male farmer presented to a rural Tanzanian referral hospital with 1 week of worsening generalized abdominal pain, exacerbated by food intake. For 2 years, he had experienced intermittent epigastric and upper abdominal discomfort, bloating, occasional vomiting, substantial unintentional weight loss, and recurrent night sweats. A sibling had been treated for pulmonary tuberculosis in 2019. He had no previous medical or surgical history, used no regular medication, and reported no drug allergies. He was cachectic but alert and haemodynamically stable, with generalized tenderness, guarding, and rebound tenderness. Laboratory testing showed leukopenia, raised C-reactive protein, and mild renal impairment. Chest radiography demonstrated free subdiaphragmatic air without radiographic evidence of active pulmonary tuberculosis.

Emergency laparotomy revealed several litres of enteric fluid and a large pyloroduodenal perforation. The distal stomach, pylorus, and proximal duodenum were markedly thickened, oedematous, friable, and anatomically distorted by severe chronic inflammation superimposed on delayed perforation. Minimal mobilization required to define the lesion produced further disruption and demonstrated near-complete structural loss of the segment. Primary closure, omental patch repair, pyloric exclusion, and limited ulcer surgery were considered but were unsafe because insufficient viable tissue remained. Distal gastrectomy, closure of the remaining viable distal duodenum, and Roux-en-Y gastrojejunostomy were therefore performed for source control. The first specimen was inadvertently lost before histopathological processing.

The initial postoperative course appeared satisfactory, but persistent high-output clear drainage developed, reaching ~1.5 l/day without visible bile. Drain amylase measurement was unavailable, although the volume and appearance suggested pancreatic secretion. Progressive jaundice then developed. Ultrasonography showed no biliary dilatation, but the combination raised concern for continuing pancreaticoduodenal disruption with impaired distal biliary drainage. Relaparotomy on postoperative Day 8 demonstrated extensive destruction of the second duodenum and surrounding pancreaticoduodenal tissues. Distal biliary continuity was no longer identifiable in the densely inflamed field, and the process extended deeply into the pancreatic head region. Local duodenal repair, isolated biliary reconstruction, or drainage alone could not address the combined failure.

**Figure 1:**
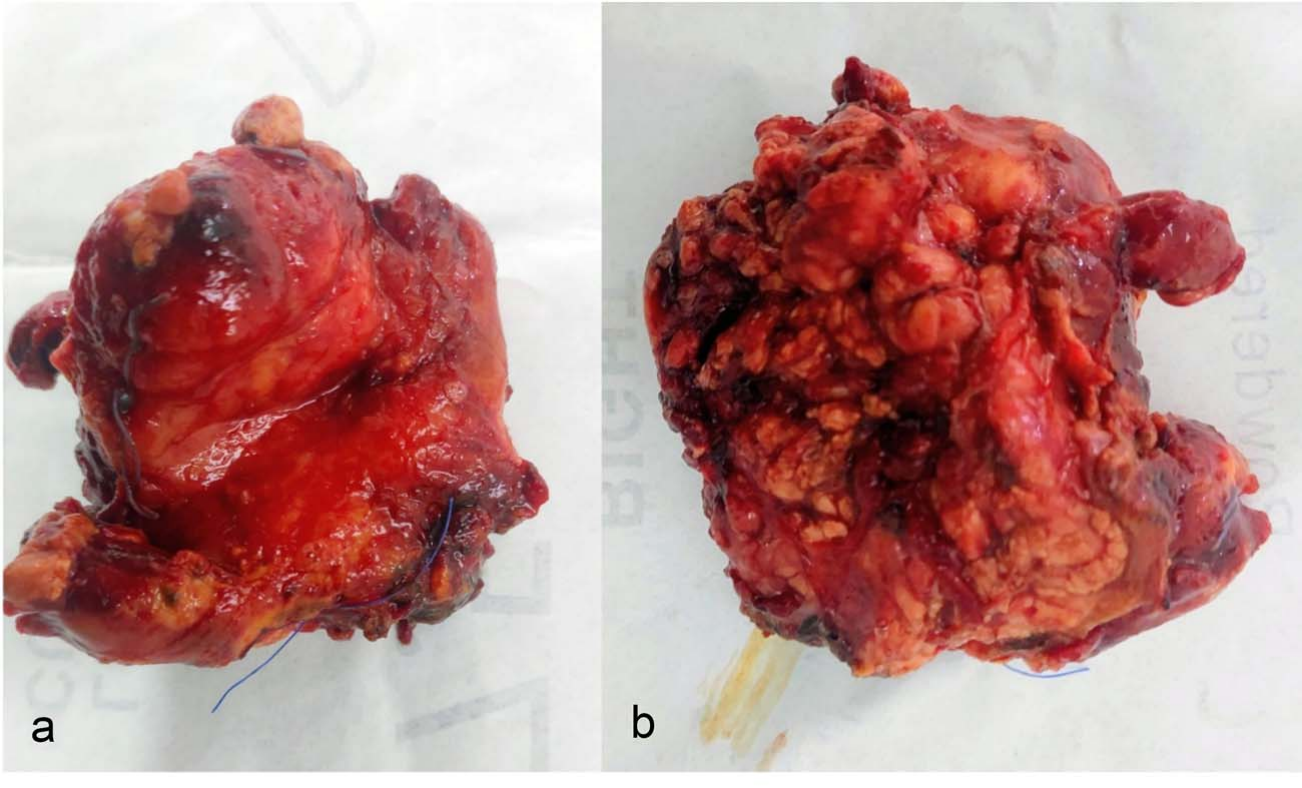
Gross specimen after salvage pancreaticoduodenectomy; (a) duodenal aspect showing an ulcerated penetrating lesion subsequently demonstrated histologically to contain necrotizing granulomatous inflammation; (b) posterior pancreatic aspect showing the irregular operative resection surface.

A tailored salvage pancreaticoduodenectomy was performed without external specialist support but with senior expertise in complex upper abdominal surgery. A small portion of the uncinate process densely adherent to the superior mesenteric vein was preserved to avoid vascular injury; the viable third and fourth portions of the duodenum and previous gastrojejunostomy were retained. The specimen showed the ulcerated duodenal lesion and the irregular posterior operative resection surface (Fig. 1).

**Figure 2:**
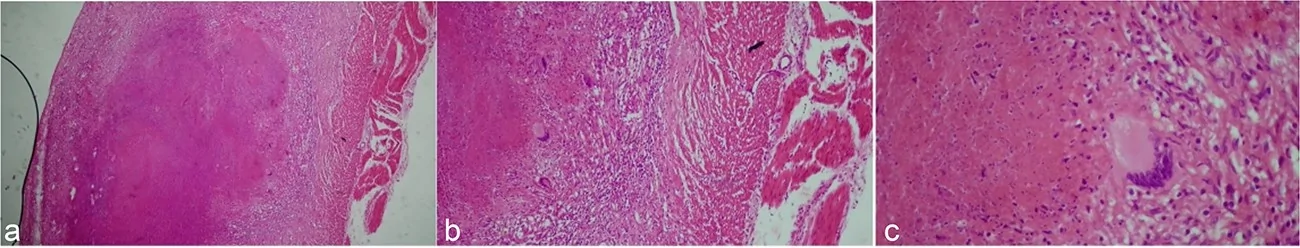
Histopathological features of duodenal tuberculosis; (a) low-power haematoxylin and eosin section showing extensive necrotizing granulomatous inflammation involving the duodenal wall; (b) intermediate-power view showing epithelioid histiocytes and multinucleated giant cells extending into the muscularis propria; (c) high-power view showing a Langhans-type giant cell adjacent to necrotic material.

Reconstruction used a second Roux limb with pancreaticojejunostomy and hepaticojejunostomy, plus controlled T-tube biliary drainage. The gland was soft and the main pancreatic duct was not identifiable, so the jejunal opening was aligned with the transection surface. Histology demonstrated extensive necrotizing granulomatous inflammation with epithelioid histiocytes and Langhans-type giant cells throughout the duodenal wall, extending into peripancreatic fat and gallbladder serosa while sparing pancreatic acini and gallbladder mucosa (Fig. 2).

Stool GeneXpert was negative, Ziehl–Neelsen staining was recommended but microbiological confirmation was unavailable, *Helicobacter pylori* testing was positive, and human immunodeficiency virus serology was negative. Antituberculous treatment was initiated. A clinically suspected pancreatic leak resolved conservatively, allowing drain removal on postoperative Day 23. The patient was discharged on Day 28 and remained well at 3 months, when the T-tube was removed.

## Discussion

The pathological distribution supported contiguous spread from destructive duodenal disease rather than primary pancreatic or gallbladder tuberculosis. The combination of perforation, peripancreatic and gallbladder serosal extension, pancreaticoduodenal disruption, and loss of biliary continuity was not described in the perforated duodenal tuberculosis reports identified (Table 1). It also differs from pancreaticoduodenectomies undertaken because tuberculosis formed a mass and mimicked a periampullary or pancreatic tumour [7–10].

**Table 1:** Published perforated duodenal tuberculosis reports compared with the present case

Report	Surgery	Pancreaticobiliary findings
Berney *et al*. [5]	Local repair	No destructive adjacent involvement reported
Qureshi *et al*. [6]	Emergency surgery	No destructive adjacent involvement reported
Talbi *et al*. [4]	Ulcer excision and repair	No destructive adjacent involvement reported
Present case	Distal gastrectomy followed by salvage pancreaticoduodenectomy	Peripancreatic and gallbladder serosal extension with loss of distal biliary continuity

At the first operation, the immediate problem was loss of viable pyloroduodenal tissue in a contaminated abdomen; source-control decisions therefore depended on tissue viability and the anatomy encountered rather than a known aetiology [11]. At relaparotomy, combined duodenal, pancreatic, and biliary disruption precluded a limited reconstruction. Emergency pancreaticoduodenectomy carries substantial risk but may be lifesaving when complex non-traumatic pancreaticoduodenal failure cannot be controlled by lesser procedures [12]. It should not be interpreted as a general treatment for duodenal tuberculosis.

Important limitations are the loss of the first specimen, absent microbiological confirmation, unavailable drain amylase, and concomitant *H. pylori* positivity. An iatrogenic contribution to distal biliary obstruction or injury during the first operation cannot be excluded because the anatomy was profoundly altered by inflammation and contamination. Nevertheless, at relaparotomy the extensive destruction made local repair or isolated biliary reconstruction unfeasible regardless of the relative contribution of disease progression or operative injury. In tuberculosis-endemic settings, histopathological assessment is essential when young patients with constitutional symptoms and relevant exposure present with unexpectedly destructive duodenal perforation. Diagnostic tissue should be preserved whenever possible, and salvage surgery should be tailored to the viable anatomy rather than generalized from this exceptional outcome.

## Supplementary Material

CAREChecklistJSCR_rjag801

## Data Availability

All data supporting this case report are included in the article.

## CARE checklist

This case report was prepared in accordance with the CARE guidelines [13]. The completed CARE checklist is submitted as a separate file.
