# Supplementary material for: Perforated duodenal tuberculosis with pancreaticobiliary disruption requiring salvage pancreaticoduodenectomy: a case report
Source: J Surg Case Rep. 2026 Sep 3;2026(9):rjag801. doi: 10.1093/jscr/rjag801 (PMC13540722; doi:10.1093/jscr/rjag801)
Supplement: Supplementary file 1 [file carechecklistjscr_rjag801.docx]

CARE Checklist

Manuscript: Perforated Duodenal Tuberculosis with Pancreaticobiliary Disruption Requiring Salvage Pancreaticoduodenectomy: A Case Report

| **Topic** | **Item** | **Checklist requirement** | **Reported in manuscript** |
| --- | --- | --- | --- |
| Title | 1 | Diagnosis/intervention followed by “case report”. | Title |
| Keywords | 2 | Two to five key words including “case report”. | Abstract page, Keywords |
| Abstract | 3a-d | Uniqueness, symptoms/findings, diagnosis/interventions/outcome, and take-away lesson. | Abstract |
| Introduction | 4 | Why the case is distinctive, with relevant literature. | Introduction |
| Patient information | 5a-d | De-identified demographics, symptoms, history, and previous interventions. | Case Report, paragraph 1 |
| Clinical findings | 6 | Relevant examination and clinical findings. | Case Report, paragraph 1 |
| Timeline | 7 | Chronological clinical course. | Case Report, paragraphs 1-4 |
| Diagnostic assessment | 8a-d | Testing, diagnostic challenges, diagnosis, and differential diagnosis. | Case Report, paragraphs 1 and 4; Discussion |
| Therapeutic intervention | 9a-c | Surgical and pharmacological interventions and changes with rationale. | Case Report, paragraphs 2-4 |
| Follow-up and outcomes | 10a-d | Outcomes, adherence/tolerability, and adverse events. | Case Report, paragraph 4 |
| Discussion | 11a-d | Strengths/limitations, literature, rationale, and take-away lesson. | Discussion |
| Patient perspective | 12 | Patient perspective, when available. | Not separately reported; patient satisfaction and adherence were documented in the source record. |
| Informed consent | 13 | Written informed consent. | Patient Consent statement |
